# Influence of Implant Connection, Abutment Design and Screw Insertion Torque on Implant-Abutment Misfit

**DOI:** 10.3390/jcm9082365

**Published:** 2020-07-24

**Authors:** Jorge Vélez, Jesús Peláez, Carlos López-Suárez, Rubén Agustín-Panadero, Celia Tobar, María J. Suárez

**Affiliations:** 1 Department of Conservative Dentristy and Bucofacial Prosthesis, Faculty of Odontology, University Complutense of Madrid, 28040 Madrid, Spain; pipo_velez@hotmail.com (J.V.); carlop04@ucm.es (C.L.-S.); celiatobar@gmail.com (C.T.); mjsuarez@ucm.es (M.J.S.); 2 Department of Dental Medicine, Faculty of Medicine and Dentistry, University of Valencia, 46010 Valencia, Spain; ruben.agustin@uv.es

**Keywords:** external connection, internal connection, abutment, implant-abutment interface, misfit

## Abstract

Background: An accurate fit at the implant-abutment interface is an important factor to avoid biological and mechanical complications. The aim of this study was to evaluate the marginal misfit at the implant-abutment interface on external and Morse taper connection, with straight and angulated abutments under different insertion torque loads. Materials and Methods: A total of 120 implants were used, 60 with external connection (EC) and 60 with Morse taper connection (IC). Straight (SA) (*n* = 60) and angulated abutments (AA) (*n* = 60) were randomly screwed to each connection at different torque levels (*n* = 10 each): 10, 20 and 30 Ncm. All specimens were subjected to thermal and cyclic loading and the misfit was measured by scanning electron microscopy. Data were analyzed with one-way ANOVA, *t*-test and Kruskal-Wallis test. Results: Significant differences (*p* < 0.001) were found between connections and abutments regardless of the torque applied. Morse taper connections with straight and angulated abutments showed the lowest misfit values (0.6 µm). Misfit values decreased as torque increased. Conclusions: The misfit was affected by the type of connection. The type of abutment did not influence the fit in the Morse taper connection. The higher the tightening torque applied the increase in the fit of the implant-abutment interface.

## 1. Introduction

During the last decade, dental implants have been constantly evolving through development and research in order to improve the quality of patient care, allowing us to practice a comprehensive and global restorative dentistry, which means obtaining a complete integration between the hard and soft peri-implant tissues [1]. Osseointegration has been considered as a fundamental and priority factor related to the success of the implants [2,3]. However, biological complications can occur due to the bacteria penetration into the microgap at the implant-abutment interface [4,5].

Since the introduction of dental implants, several implant-abutment connection designs have been developed [6]. The first osseointegrated implants had an external hexagon design on the implant platform [7]. This type of connection has been associated with a certain amount of peri-implant bone loss, especially during the first year of performance [8,9]. Such bone loss may be due to chronic inflammation in the implant-abutment interface, the distribution of tensions in the marginal bone crest and the presence of micromovements in the implant-abutment interface. [1,10,11]. To overcome some of the design limitations and bone loss of the external hexagonal connection, internal connection with a wide variety of shapes was introduced [12]. Internal connections provide better esthetics, better joint strength, an improved microbial seal, better long-term stability of the implant-abutment complex and better crestal bone levels in the short-medium term as compared to external connection [9,12,13]. Morse taper connection is a conical internal connection that creates a friction between the surfaces that result in cold-welding [14,15]. This connection provides a larger surface area of the implant-abutment interface compared to parallel walled connection producing a good seal between its components and therefore less microleakage. In addition, it also produces superior joint stability and less marginal bone loss [5,12,15,16,17,18].

Regardless of the type of geometrical configuration of the implant, the prosthetic abutment will be fixed to the implant through a screw, generating an interface between the implant-abutment junction [19]. The unavoidable gap between the implant and the abutment may cause biological complications due to the passage of bacteria and/or their metabolic products towards the connection [20]. These bacteria and their metabolites act directly on the peri-implant tissues and cause inflammation and bleeding, originating in an irreparable damage to the peri-implant tissues, with subsequent bone loss and the implant itself. [4,10,14,20,21,22,23]. Furthermore, the presence of the gap can incorrectly transmit the forces from the abutment to the implant, generating constant micromovements, which over time can cause biomechanical complications, such as: abutment screw loosening, rotation and/or fracture of the screw or the abutment, and a reduction in the prosthetic screw preload [24,25,26].

The degree of filtration between and implant and its prosthetic components depends on variable factors, such as the geometry of the connection, a precise fit between the components, the rotational freedom of the abutment on the implant, the applied torque load to tighten the abutment, the micromovements between the components of the implant-abutment complex during function and the abutment materials [13,24,25,27].

Although there are several studies comparing the marginal fit of different geometry connections with different abutment materials, limited data exist on the influence of the different torque levels on the fit of the implant-abutment interface and there is a lack of studies comparing straight and angulated abutments. The present study aimed to evaluate the marginal misfit of the implant-abutment interface on external and Morse Taper connection, with straight and angulated abutments and at different insertion torque loads. The null hypothesis tested was that there would be no differences in the misfit between implants with external and internal connection, with straight and angled abutments and with different insertion torque loads applied.

## 2. Material and Methods

### 2.1. Implants and Abutments

Two different implant designs with different connections were used in the study: external hexagon connection (*n* = 60) (EC) and internal connection with 11° Morse taper double internal hexagon with platform switching (*n* = 60) (IC). Both types of implants (*n* = 120) had the same dimensions (Table 1). Two types of abutments were used for both implant connections: straight hexagonal abutment (*n* = 60) (SA) and a 15° angulated abutment (*n* = 60) (AA). The cuff height was 2 mm in both types of abutments (Table 2).

### 2.2. Preparation of Specimens and Placement of Implant and Abutments

One hundred and twenty specimens (10 mm high, 15 mm wide and 15 mm deep) were manufactured in machined methacrylate. A central perforation with a diameter of 3.75 mm was carried out in the specimen in order to place the implants on the same vertical axis and to avoid the angulation of the implants and abutments. To ensure that all implants were placed in the same central point of the specimen, a parallelizing machine was used with the surgical kit of the company (Mozo-Grau SA, Valladolid, Spain), simulating the surgical perforation in the bone. The implants were placed with a standard drilling sequence at 800 rpm and at a torque of 35–45 Ncm as recommended by the manufacturer.

The abutments were randomly screwed into their respective implants by the same operator, manually using a 1.25 mm hexagonal screwdriver (Mozo-Grau SA). Subsequently, 30 SA and 30 AA, were screwed for both connections. All the abutments were placed by applying 10, 20, and 30 Ncm torque load in each connection group with a dynamometric torque wrench (Mozo-Grau SA), so that the procedure can be comparable with the clinical screwing of the abutments in the mouth. The abutments were retightened after 10 min with their respective torque, as previously reported [17,28]. The torque applied to the abutments was always made by the same operator.

Four groups (*n* = 30 each) were created, according to the type of connection and abutment. Each group was randomly divided into three subgroups (*n* = 10 each), according to the torque applied to the abutments (10, 20, 30 Ncm). The groups analyzed are summarized in Table 3. Power analysis was carried out, concluding that a minimum of seven specimens per group were needed to achieve a power of 80%, for 95% confidence.

### 2.3. Thermocycling and Cyclic Loading

A cylindrical base was fabricated of hard plastic to position the specimens in the chewing simulator (4.5 mm diameter, 2.9 mm height). Subsequently, a condensation silicone guide (Lab-Putty; Coltène, Altstätten, Switzerland) was placed with the shape of the specimens inside the plastic base, with the aim of placing the specimens in the same central position. Once the specimens were positioned, the base was backfilled with a self-curing epoxy resin GIV (Polipox, Sao Paulo, Brazil), with a modulus of elasticity greater than 3 GPa that allows for the simulation of the conditions where the bone absorbs the forces. Each specimen was placed 3 mm above the resin, simulating 3 mm of bone resorption according to the specifications detailed in the ISO 14801:2007 standards [29]. All specimens were stored inside 40 cylindrical polyethylene jars (Resopal, Madrid, Spain), filled with 40 mL of Fusayama-Meyer artificial saliva (LCTech, Obertaufkirchen, Germany). The chemical composition of the artificial saliva is shown in Table 4.

The specimens were subjected to thermal cycling for 1200 cycles, alternating the temperature between 5–55 °C using a climate chamber (CCK 40/81, Dycometal, Viladecans, Spain) controlled with Eurotherm iTools software (Eurotherm, Worthing, UK). All specimens underwent 250,000 mechanical cycles performed by a chewing simulator (Chewing Simulator CS-4.2 economy line, SD Mechatronik GmbH, Feldkirchen-Westerham, Germany). A vertical load of 200 *n* with vertical (2.5 mm) and lateral displacement (0.7 mm) at 60 mm/s crosshead speed was performed in the center of the abutments at a frequency of 2 Hz. After cyclic loading, the specimens were embedded in round molds with an epoxy resin (Aka-Resin and Aka-Cure, Akasel, Roskilde, Denmark). Subsequently, they were worn with a flat surface grinding machine until reaching the central axis of the implant (Schaublin 102VM, Switzerland). The final finishing and polishing of each specimen were carried out with silicon carbide sandpaper of different grains (P320, P600, P1200 and P4000; 3M, St Paul, MN, USA) and chemical polishing with silica colloidal suspension (Akasel).

### 2.4. Implant-abutment Misfit Evaluation

**Figure 1:**
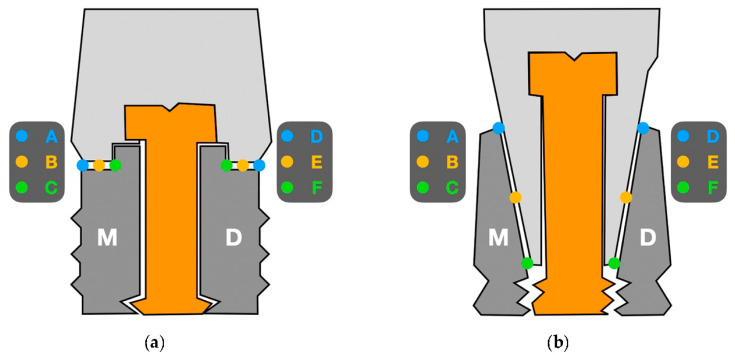
Scheme of the positions used for measuring the implant-abutment interface: (**a**) external connection, (**b**) Morse taper connection.

**Figure 2:**
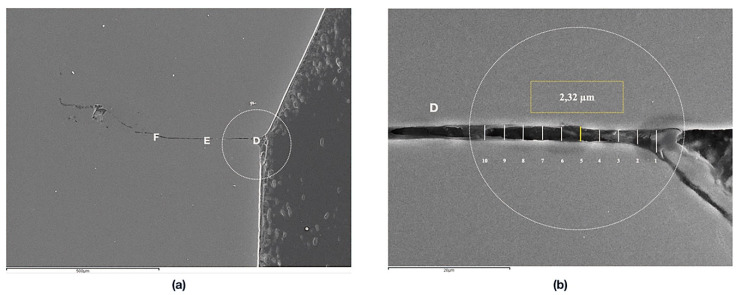
SEM images (1000×): (**a**) Reference points for the measurements in the external connection, (**b**) Detail of an edited image increasing the number of measurements per point.

**Figure 3:**
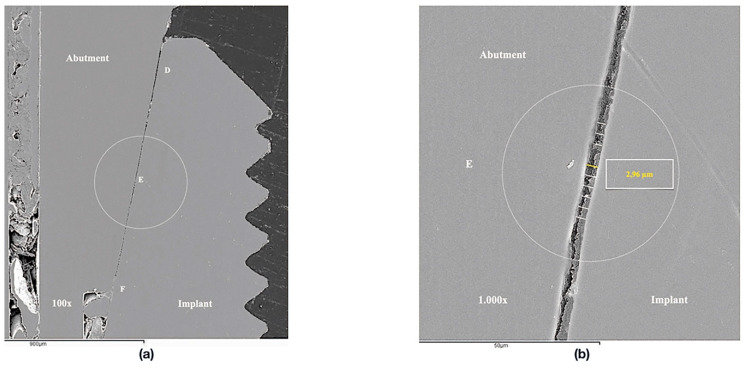
SEM images (1000×): (**a**) Reference points for the measurements in the Morse taper connection. (**b**) Detail of an edited image increasing the number of measurements per point.

Implant-abutment interface adjustment was performed under a scanning electron microscope (SEM) (JSM 6400, JEOL, Tokyo, Japan). Before the SEM evaluation, the samples were coated with 24 kt, 19.32 g/m^3^ density gold by a metallizer (Q15rs, Quorum Technologies, Sussex, UK). The images were captured in the mesial and distal area of every specimen by the software INCA suite 4.04 (Oxford Instruments, Abingdom, UK) with a 1000× magnification approach. The total length of the implant-abutment interface was divided in three equidistant points with a separation range of 3 µm, on both sides, in order to ensure uniform measurements at the same points in all specimens. Subsequently, the measurements were performed on the equidistant points, which were defined as A, B, C on the mesial area, and D, E and F on the distal area (Figure 1). The images obtained were edited using the ImageJ V.1.51 software (National Institutes of Health, San Antonio, TX, USA) to increase the number of measurements per specimen by producing lines parallel to the original image, and ten measurements per each point were registered (Figure 2 and Figure 3). Therefore, 60 measurements per specimen were recorded. The measurements were taken by two independent researchers (JV and JP).

### 2.5. Statistical Analysis

Means and standard deviations (SD) per group were calculated. One-way analysis of variance (ANOVA), Tamhane T2 post hoc test and Student *t* test, was performed to compare connections and abutments regardless of the torque load applied. Additionally, given the non-normality of data on torque load and misfit, the comparisons among the groups were performed with the Kruskal-Wallis test followed by a post-hoc multiple comparison test with Bonferroni correction. Statistical analysis of all variables was performed with the SPSS 22.0 software (SPSS Inc, Chicago, IL, USA). The level of significance was established at α = 0.05.

## 3. Results

All groups analyzed showed misfit values below 3 µm, regardless of the connection, abutment or torque load. The means and SD for misfit values of both connections, both abutments and the different torque loads applied are presented in Table 5, Table 6 and Table 7.

Implant-abutment connections significantly affect the marginal misfit (*p* = 0.001), regardless of the abutment and torque load, showing, in the *t* test, that the EC presented higher marginal misfit than the IC (Table 5). Likewise, significant differences were also found between both abutments (*p* = 0.004) regardless of the connection and torque load, demonstrating SA had a better marginal fit than AA (Table 5). Comparisons of the marginal discrepancies in both connections with both abutments, regardless of torque load, by ANOVA revealed significant differences among the groups (*p* = 0.001). The EC-SA group presented a lower value of marginal misfit than the EC-AA, with significant difference (*p* = 0.005). Likewise, significant differences were shown for the EC with both abutment groups compared to IC, except for the EC-SA and IC-AA groups. No differences were observed between both abutments for the IC. The IC-SA showed the lowest misfit values (Table 6).

**Figure 4:**
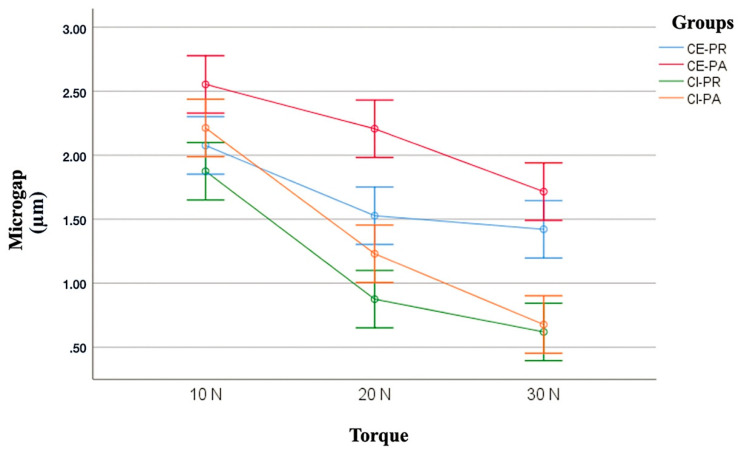
Box plot of misfit values (µm) among the groups according to the applied torque.

When analyzing the marginal discrepancy among the different torque loads applied (10, 20, 30 Ncm), regardless of the connection and abutment, the Kruskal-Wallis test indicated that significant differences were observed (*p* = 0.001). The 30 Ncm torque load showed the best marginal fit compared to the 10 and 20 Ncm torque loads. Significant differences were observed between the 10 and 20 Ncm loads (*p* = 0.001), and the 10 and 30 Ncm loads (*p* = 0.001), but no significant differences were observed between the 20 and 30 Ncm loads (*p* = 0.10). The Kruskal-Wallis test showed no significant differences among the groups with the 10 Ncm load applied. However, significant differences were observed for the 20 Ncm load (*p* = 0.001) between both connections with AA (*p* = 0.002), and for the 30 Ncm load (*p* = 0.001) between both connections with SA and AA (*p* = 0.005 and *p* = 0.001, respectively) (Table 7). The different torque loads applied did not affect the marginal fit of the ECSA group. Nevertheless, significant differences were observed for the ECAA group between the 10 and 30 Ncm loads (*p* = 0.008). Likewise, significant differences were shown for the ICSA (*p* = 0.001) and ICAA groups (*p* = 0.001). The post-hoc test indicated differences between the 10 and 20 Ncm loads (*p* = 0.006), and between the 10 and 30 Ncm loads (*p* = 0.001) for the ICSA group. In the ICAA group, differences were also observed between the 10 and 20 Ncm loads (*p* = 0.02) and between the 10 and 30 Ncm loads (*p* = 0.001). The lowest marginal misfit values were observed with the 30 Ncm torque load for all the groups analyzed. (Table 7 and Figure 4).

## 4. Discussion

The present study attempts to evaluate and to compare the misfit at the implant-abutment interface in two types of connections (external and internal), with two types of abutments (straight and angulated), at different insertion torque loads. The results obtained support the rejection of the null hypothesis, as differences in marginal adjustment were found depending on the type of connection, the type of abutment and the different tightening torque load.

The precision of fit at the implant-abutment interface is a very important criterion for the long-term osseointegration and success of dental implants [6]. The misfit at the implant-abutment interface can produce biological (microbial colonization, bone loss and loss of implant osseointegration) and mechanical complications (screw loosening/fracture, abutment fracture) [5,10,12,20,21,30,31,32]. The presence of micro gap at the implant-abutment connection depends on several factors, such as the imprecise mechanization of the implant connection, inadequate implant-abutment adaptation or the torque load applied [10,17,31].

The results of the present study revealed very low misfit values in both connections, regardless of the type of abutment or torque load applied, presenting the internal connection with the lowest misfit values. The external hexagon connection analyzed showed much lower misfit values than previous studies [6,11,24,30,33,34]. Regarding the Morse taper connection, several previous studies reported misfit values higher (2.3–5.6 µm) than in the present study (1.25 µm) [17,27,33], while other studies reported similar results [11]. It has been previously reported that all implants present a gap at the implant-abutment interface [4,10,11], and in the study both connections analyzed present a slight misfit. Internal connection has been reported to be superior to an external one regarding the long-term stability of the implant-abutment complex [35]. Some studies reported larger discrepancies for external connection compared to internal connection [6,9,11,33,36], while others reported superior marginal fit for external connection [13]. Furthermore, conical connection has demonstrated better fit and stability than non-conical connection [9,18]. Morse taper connection presents positive geometric locking, based on a friction mechanism that creates a wedging effect and a hermetic seal that protects the abutment screw from excessive functional load, reducing the biological and mechanical complications [9,12,17,27,37]. The best results for Morse taper connection could be explained due to the length of the implant-abutment connection and the precise adaptation in the deeper inner portions of the system, resulting in reduced micromovements and superior torque maintenance and abutment stability [5,18,38,39]. The low values found in the study for both connections could be due to the precise manufacturing criteria followed by the manufacturer.

There is a great variability in the marginal misfit values reported in previous studies that could be due to the different methodologies and terminology used. Although a classification has been proposed to measure the implant-abutment gap [30], other studies evaluated the gap using Holmes’ adapted terminology [13,33], and others use their own terminology [6,11,17]. Therefore, there is no consensus on studies and comparisons among them are difficult. Differences were also observed regarding the methodology to evaluate the implant-abutment interface. Some of the studies used the technique of direct fit measurement at the implant-abutment interface in non-sectioned specimens [6,24,40], or sectioned specimens [11,13,17,33], although there are variations in the measurement instruments—optic microscope [30], stereosmicroscope [40], SEM [6,11,12,17,24], scanning laser microscope [33] or video measuring machine [13]—while other studies evaluated the microleakage [5,11,14,19,36,41] or the abutment micromotion [12]. In the present study, direct observations of the interface in sectioned specimens were performed under SEM. Nowadays, universally accepted methods for testing the implant-abutment interface fit do not exist; therefore, it is difficult to make comparisons among the studies [12]. Another factor that can influences the results of the studies is the conditions of the experiments. A few in vitro studies, especially the most recent, have performed cyclic loading in an attempt to simulate clinical conditions to assess the implant-abutment connection [11,14,28,34,36,41,42]. In the study the specimens were subjected to thermal and mechanical cycles simulating the loading conditions of clinical environment.

Regarding the abutments, previous studies analyzed the implant-abutment misfit in straight abutments, comparing different materials (metal-based or zirconia especially) or fabrication techniques (prefabricated or customized) [4,6,13,42,43,44,45]. The results showed better fit in titanium compared to zirconia abutments [6,13,40] and in prefabricated abutments [4,13,42,45]. Regarding the type of titanium employed for implant abutments, the literature is sparse and focuses mainly on mechanical properties. Titanium grade V demonstrated superior strength, a smaller frictional coefficient, higher preload and higher rotational misfit compared to titanium grade IV specimens [46,47,48]. These characteristics may diminish screw loosening under functional load specimens [49]. Furthermore, the use of abutment materials with high strength and low frictional coefficients has been recommended to improve the mechanical stability of the implant-abutment connection [47]. In the study, the abutment material was titanium grade V and could have contributed to the low misfit values found.

Likewise, there are very few studies that analyzed angulated abutments compared to straight abutments, indicating that the straight abutment has better prognosis and less mechanical failures [50]. However, to the best of our knowledge, there are no previous studies analyzing the angle of the abutment and its relationship with the misfit of the implant-abutment interface in different types of connections. Therefore, it is impossible to compare the results of the study. The results showed differences between the straight and angulated abutments in the external connection, but no differences were observed for the internal connection. The straight abutments have a better marginal adjustment of the interface than the angulated one in both connections regardless of the applied torque load, although the misfit values obtained were very low (1.3 and 2.1 µm, respectively). These results can be explained because, due to the angle of the abutment, the loads do not follow the longitudinal axis of the implant-abutment complex, generating a greater amount of micromovement, which can produce a worse transmission of forces. Furthermore, as the specimens were subjected to cyclic loading, increased surface wear due to friction of the components could occur in the angulated abutments, resulting in a lower adjustment and a larger size of the misfit at the implant-abutment interface.

On the other hand, a close relationship was observed between the tightening torque of the prosthetic abutment screw and the adjustment at the interface. The lowest misfit values were obtained when the torque was increased. The results are consistent with previous studies reporting that the linear area of contact between the abutment and the implant increased as the torque increased [6,11,16,17,40]. When torque is applied, the screw is elongated, causing stress on the stem and threads and creating a compressive strength that holds the implant-abutment connection together [17,51,52]. Furthermore, the degree of settling depends on the magnitude of the tightening torque [49]. The decrease in the misfit as torque increases could be explained because, when torque increases, greater compression is generated between both surfaces, providing greater stable connection [17,52,53].

The best fit values were obtained for the Morse taper connection at 30 Ncm torque load in both abutments, which is the manufacturer´s recommended torque, with values close to 0 µm. These results have clinical relevance, indicating that it is very important to follow the manufacturer´s instructions. However, no differences were observed between 20 and 30 Ncm, as previously reported [18]. There may be complications as a result of an inadequate torque load. When the abutments are tightened with forces lower than those recommended by the manufacturer, the risk of screw loosening is high [53,54,55]. In addition, screw fractures and deformations in the implant and screw joints have been reported under a low preload force [56]. Furthermore, low torque values reduce the amount of contact between implant-abutment connection, resulting in less effective bacterial seals and more microleakage [4,11,17]. On the other hand, when the screw is tightened with forces greater than those recommended, the screw exceeds its yield strength, loosening its mechanical characteristics [54,57]. This effect leads to a decrease in preload and screw fracture can happen [53,57,58]. Therefore, it is important to make use of the adequate tightening torque for clinical success [56].

The study had several limitations. Only one implant system was analyzed, and it would be interesting to compare the results with other implant systems from other manufacturers and with different geometry implant connections and abutments materials. In addition, the study did not address the microleakage and its relationship with the fit. However, the low misfit values found may indicate that the bacterial leakage could be reduced. Furthermore, there is a need to stablish a standardized method to assess marginal fit of the implant-abutment interface.

## 5. Conclusions

Within the limitations of this study, implants with Morse taper connection had better marginal adjustment than external connection regardless of the torque or the type of abutment. Straight abutments demonstrated less misfit that the angulated abutments in both connections. The misfit is directly proportional to the applied torque, with lower misfit values at higher insertion torque.

## Figures and Tables

**Table 1:** Characteristics of the implants used.

Implant	Connection	Dimensions	Platform	Material	Manufacturer
MG-Osseous Standard	External hexagon	3.75 × 11.5 mm	4.1 mm	Titanium Grade IV	Mozo-Grau SA, Valladolid, Spain
MG-Standard InHex	11° Morse taper	3.75 × 11.5 mm	2.8 mm	Titanium Grade IV	Mozo-Grau SA

**Table 2:** Characteristics of the abutments used.

Abutment	Connection	Platform	Diameter	Material	Manufacturer
Standard MG-Osseous	External	4.1 mm	4.8 mm	Titanium Grade V	Mozo-Grau SA, Valladolid, Spain
Standard MG-Inhex prepable	Morse Taper	2.8 mm	4 mm	Titanium Grade V	Mozo-Grau SA
Standard MG-Osseus 15°angled	External	4.1 mm	4.8 mm	Titanium Grade V	Mozo-Grau SA
Standard MG-Inhex 15° angled prepable	Morse Taper	2.8 mm	4 mm	Titanium Grade V	Mozo-Grau SA

**Table 3:** Classification of the tested groups, according to the connection, the abutment and the torque load (Ncm) (EC: external connection; IC: internal connection; SA: straight abutment; AA: angulated abutment).

Test Groups	Connection	Abutments	Torque
EC-SA	External	Straight	10
			20
			30
EC-SA	External	Angulated	10
			20
			30
IC-SA	Morse taper	Straight	10
			20
			30
IC-AA	Morse taper	Angulated	10
			20
			30

**Table 4:** Composition of artificial saliva.

Chemical Product	Composition (g/dm)^3^
K_2_HPO_4_	0.20
KCL	1.20
KSCN	0.33
NA_2_HPO_4_	0.26
NACL	0.70
NAHCO_3_	0.50
Urea	1.50
Lactic acid	PH greater than 6.7

**Table 5:** Means and standard deviation (SD) misfit values (micrometers) for connections regardless of abutments and torque, and abutments regardless of connection and torque (EC: external connection; IC: internal connection; SA: straight abutment; AA: angulated abutment).

Type	Group	*n*	Mean	SD
Connection	EC	60	1.92	0.57
	IC	60	1.25	0.65
Abutment	SA	60	1.40	0.62
	AA	60	1.7	0.74

**Table 6:** Means and standard deviation (SD) misfit values (micrometers) for connections and abutments regardless of torque load (EC: external connection; IC: internal connection; SA: straight abutment; AA: angulated abutment).

Connection	Abutment	*n*	Mean	SD
EC	SA	30	1.67	0.52
	AA	30	2.16	0.53
IC	SA	30	1.12	0.59
	AA	30	1.37	0.71

**Table 7:** Means and standard deviations (SD) misfit values (micrometers) for each group according to the tightening torque applied (EC: external connection; IC: internal connection; SA: straight abutment; AA: angulated abutment).

Torque	Group	*n*	Mean	SD
10 Ncm	EC-SA	10	2.07	0.41
	EC-AA	10	2.55	0.47
	IC-SA	10	1.87	0.24
	IC-AA	10	2.21	0.23
20 Ncm	EC-SA	10	1.52	0.33
	EC-AA	10	2.20	0.30
	IC-SA	10	0.80	0.10
	IC-AA	10	1.23	0.45
30 Ncm	EC-SA	10	1.42	0.57
	EC-AA	10	1.71	0.44
	IC-SA	10	0.61	0.24
	IC-AA	10	0.67	0.15
